# Investigating the Effect of Operational Variables on the Yield, Characterization, and Properties of End-of-Life Olive Stone Biomass Pyrolysis Products

**DOI:** 10.3390/molecules28186516

**Published:** 2023-09-08

**Authors:** Sina Ebrahim Atakoohi, Elena Spennati, Alessandro A. Casazza, Paola Riani, Gabriella Garbarino

**Affiliations:** 1 Dipartimento di Ingegneria Civile, Chimica e Ambientale (DICCA), Università degli Studi di Genova, Via Opera Pia 15, 16145 Genova, Italy; sinaebrahim.atakoohi@edu.unige.it (S.E.A.); alessandro.casazza@unige.it (A.A.C.); 2 INSTM, UdR Genova, Via Dodecaneso 31, 16146 Genova, Italy; paola.riani@unige.it; 3 Dipartimento di Chimica e Chimica Industriale (DCCI), Università degli Studi di Genova, Via Dodecaneso 31, 16146 Genova, Italy; 4 CNR SCITEC G. Natta, Via Golgi 19, 20133 Milano, Italy

**Keywords:** olive stone, process parameters, waste valorization, biochar, bio-oil, pyrolytic gas

## Abstract

In recent years, biomass has emerged as a promising raw material to produce various products, including hydrocarbons, platform chemicals, and fuels. However, a more comprehensive evaluation of the potential production of desirable value-added products and chemical intermediates is required. For these reasons, this study aimed to investigate the impact of various operating parameters on the pyrolysis of end-of-life olive stone, an agriculture and food industry waste, using a tubular quartz reactor operated at 773 K. The results revealed that the product compositions were comparable under batch or semi-batch nitrogen feeding conditions and with reaction times of 1 or 3 h. The product distribution and composition were significantly influenced by changes in the heating rate from 5 to 50 K min^−1^, while the effect of changing the biomass particle size from 0.3 to 5 mm was negligible in the semi-batch test. This work provides a comprehensive understanding of the relationship between pyrolysis operational parameters and obtained product distribution and composition. Moreover, the results confirmed the possible exploitation of end-of-life olive stone waste to produce high-added value compounds in the zero-waste strategy and biorefinery concept.

## 1. Introduction

The increasing energy demand, driven by industrialization and population growth, has put a strain on the world’s resources, particularly fossil fuels. The widespread use of these fuels has contributed significantly to global warming by emitting CO_2_ into the atmosphere. To address this issue, it is crucial to explore alternative energy sources that are sustainable, reliable, and environmentally friendly [1]. The adoption of a zero-waste strategy, which involves the valorization and reutilization of wastes to produce other useful products, holds great potential as a solution to several environmental challenges. This approach can help to improve energy efficiency, reduce air and water pollution, mitigate greenhouse gas emissions, and preserve natural resources [2,3,4,5,6].

Biomasses, as a natural resource, have gained considerable attention as a potential source of raw materials for various products including hydrocarbons, platform chemicals, and fuels. However, there are still significant challenges to be addressed to fully realize the potential of biomasses in meeting the global energy demand. Therefore, the functional impact of biomasses on the global energy cycle is contingent upon conducting further research to assess the practicality of their large-scale implementation and to evaluate the potential for generating valuable chemical intermediates and products [7]. The olive sector is a major contributor to the agricultural biomass waste stream. Olive stones, a byproduct of olive oil production and table olive consumption, are generated in significant quantities. Olive groves cover 11.5 million hectares, a small but significant portion of the world’s arable land. Despite the large amounts of olive stones produced by the olive oil industry, the table olive industry alone generates approximately 30,000 ton/y [8,9]. Olive stones make up 20 wt.% of olive and consist of 80 wt.% endocarp and 20 wt.% seed [10]. The usual olive stone composition is 21.9 wt.% hemicellulose, 31.9 wt.% cellulose, and 26.5 wt.% lignin. Carbon, followed by oxygen, and hydrogen to a much lower extent are the most abundant elements of olive stones. The ash content is typically less than 2 wt.% and is composed of inorganic compounds such as K_2_O, CaO, MgO, SiO_2_, Fe_2_O_3_, and Al_2_O_3_ [8,11].

Among the available thermochemical conversion technologies to convert biomass to biofuels and intermediates, pyrolysis is a promising technique owing to the production of bio-oil, biochar, and pyrolytic gas [12]. However, due to the wide range of parameters affecting the pyrolysis process and the diversity of biomass sources, significant variability in the yield and composition of the products can be expected [13]. Therefore, optimizing and adjusting process conditions to achieve the most desirable products while considering economic viability is a major challenge in pyrolysis. Key experimental parameters that influence the biomass pyrolysis process include peak temperature (highest treatment temperature) [14], gas residence time [15], type of carrier gas employed to maintain the oxygen-free conditions [16], the heating rate of the feedstock, size, and shape of biomass [12,17], absolute pressure [13,18], and biomass moisture and ash contents.

According to previous studies, an increase in peak temperature leads to a decrease in char yield and a gradual increase in fixed-carbon content in the final char [19,20,21,22,23]. Moreover, it has been observed that an increased gas residence time throughout the pyrolysis process results in a slight increase in final char yield due to a higher contact time between volatiles and solid, leading to a transition from vapor to solid carbonaceous matrix (char) phase [15]. Some researchers have investigated the impact of absolute pressure in pyrolysis, indicating that this parameter does not significantly affect the char yield, but a higher pressure slightly increases the gaseous species and decreases the liquid products [13,24,25]. Likewise, it has been reported that no change in char yield has been observed by changing the type of carrier gas from an inert one, i.e., N_2_ to CO_2,_ or a mixture of N_2_/CO_2_. However, the presence of CO_2_ in the carrier gas resulted in a substantial increase in CO concentration among gaseous products, potentially due to the occurrence of the reverse Boudouard reaction (CO_2_ + C → 2CO) and reverse Water Gas Shift (CO_2_ + H_2_ ⇄ CO + H_2_O) [25,26]. In the existing literature, some researchers have utilized an oxygen-free environment through purging, while others have employed a continuous flow of inert gas, primarily nitrogen. Particle size and heating rate have been reported to have a significant effect on product yield, with higher heating rates and smaller particle sizes being more favorable in increasing bio-oil yield [27,28]. Additionally, the mineral composition of the biomass has been found to play a crucial role in secondary pyrolysis reactions and affect the reactivity of obtained pyrolysis char. As an example, trace amounts of inorganics like K, Na, P, Ca, and Mg that are present in biomass tend to increase char and gas formation while decreasing bio-oil production [29]. Furthermore, the initial moisture content of the biomass has been found to impact both product distribution and the physicochemical properties of the bio-oil [30], with different energy demands.

Together with all the above-mentioned parameters, it is also important to take into consideration that the type of biomass feedstock employed can significantly impact the pyrolysis process, and the effects of operational conditions may vary based on the biomass type [31,32,33]. The composition of pyrolysis products is deeply dependent on the composition of the biomass and the relative mass ratios of its organic and inorganic compounds. Studies have shown that the pyrolysis of cellulose or hemicelluloses leads to a higher production of oil compared to lignin, which contributes to a greater percentage of char [29]. Consequently, it is important to consider not only the abundance and affordability of biomass but also its suitability concerning the desired goal of pyrolysis.

This work aims to valorize the end-of-life olive stone, a notably abundant form of biomass residue of the agriculture and food industry, as raw material for the thermal pyrolysis process. The primary objective of this study is to comprehensively investigate the impact of pyrolysis key process parameters—namely biomass particle size, pyrolysis time, heating rate, and the presence or absence of nitrogen as carrier gas flow (in batch and semi-batch modes)—with the dual aims of cost minimization and a thorough exploration of their influence on products distribution, composition, biochar properties, bio-oil and gas composition, as well as the determination of optimal operational conditions. This research seeks to cover all these aspects within a single comprehensive work focusing on a real end-of-life industrial product.

## 2. Results and Discussion

### 2.1. Characterization of Biomass

Olive stone particles were first characterized by measuring moisture and ash content, obtaining values of ~15 wt.% and ~1 wt.%, respectively. Moreover, fresh biomass and its ash content were separately analyzed by SEM-EDX elemental analysis and reported in Table 1. The O/C of fresh biomass ratio is 0.79 which agrees with other reports available in the literature [11], even if neither S nor N were detected in this sample while they were found in other works, with low percentages [11]. In the ashes, with the exception of oxygen, K and Ca are the most abundant elements [34]. Moreover, thanks to SEM-EDX point analysis in the ash sample, few particles containing Al were found. It is worth noting that these particles could not be identified in the overall elemental analysis of the sample due to their small amount and the instructional detection limit of 0.1 wt.%. Al-containing particles are not usually present in the initial biomass, as their content is typically quite low. These findings are consistent with previous reports, where the percentage of particles containing Al in the ash sample differs, and is attributed to differences in olive grove conditions, such as planting and harvesting methods [35,36,37], as well as the fact that the study used waste olive stones from an olive oil company, which may have unique properties.

**Figure 1:**
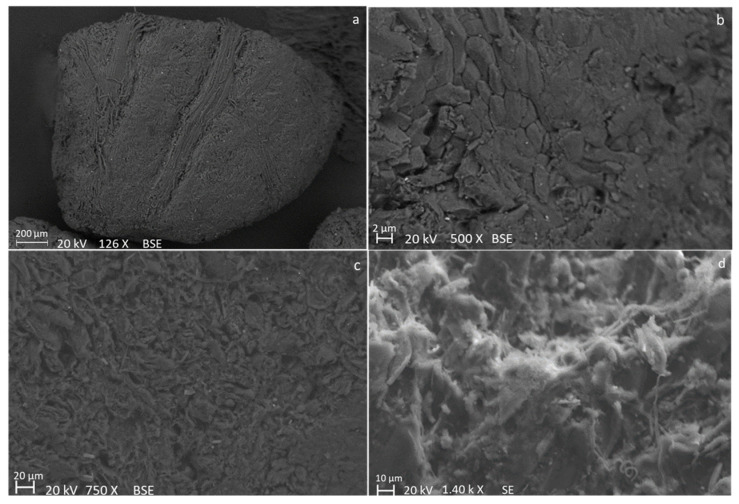
SEM images of “end-of-life” olive stone particles at different magnifications and acquired by detecting different signals: backscattered electron signal (**a**–**c**), and secondary electron signal (**d**).

SEM images of fresh olive stone (Figure 1) show a groove and porous surface. Moreover, a vascular system of fibers can be seen in Figure 1a while the parenchyma cells and plasmodesma of olive stone endocarp are observable in Figure 1b in agreement with other works [38,39].

Generally, hardness, porous matrix, high carbon, and low sulfur content make olive stone a proper precursor for further development into carbon-based materials [11,40,41].

### 2.2. Thermal Pyrolysis Results

#### Effect of Reaction Time and Nitrogen Flow Condition

**Figure 2:**
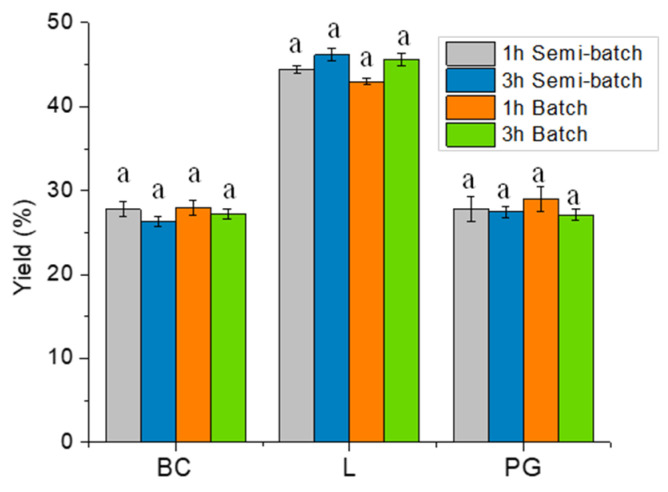
Influence of reaction time and reactor condition (batch or semi-batch) of pyrolysis test on the distribution of BC, L (HL + LL), and PG. The letter “a” within the same group indicates that there are no statistically significant differences among them.

The effect of reaction time (1 and 3 h) and N_2_ flow (batch or semi-batch conditions) on the pyrolysis products distribution is shown in Figure 2. Biochar (BC) and Liquid (L) yields were determined by gravimetric analysis and then the amount of Pyrolytic Gas (PG) was evaluated relying on total mass balances (by difference) [7]. Based on the obtained results, changing the reaction time from 1 to 3 h and the N_2_ flow conditions, the product distribution remained mainly unaffected. Nevertheless, it was noticeable that in both batch and semi-batch conditions, the amount of char slightly decreases while the amount of liquid increases with the 3 h test due to the occurrence of enhanced decomposition, in line with available data [42]. However, it is generally recognized that the effect of reaction time on biomass decomposition is most prominent during short time intervals. Moreover, the preponderance of the decomposition process takes place within the initial 30 min, according to existing literature, when considering the ramp of 50 K min^−1^ [43].

**Figure 3:**
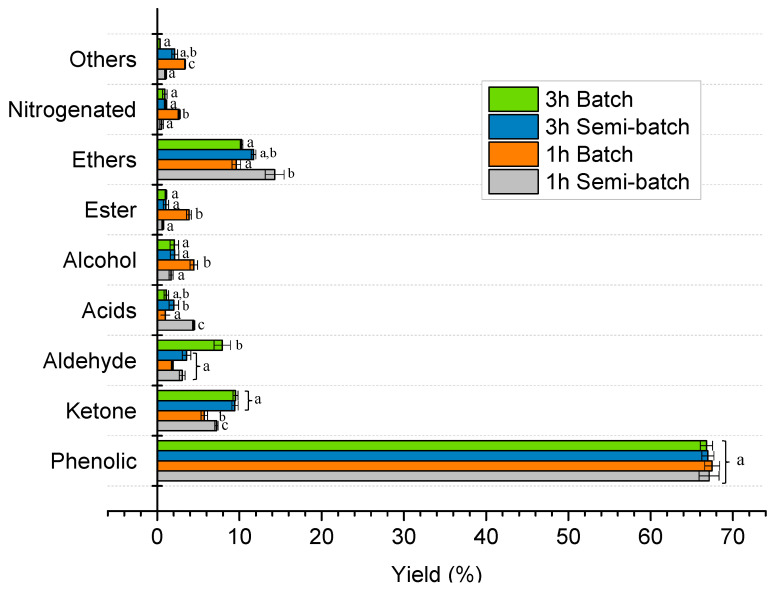
Influence of the reaction time and N_2_ feeding condition (batch or semi-batch) on the HL product distribution evaluated by GC-MS analysis. Different letters (a–c) in the same group indicate statistically significant differences among mean values.

The produced L, which has a Heavy Liquid (HL)/Light Liquid (LL) ratio of 0.53, was diluted (only HL phase) with chloroform (around 1:10) and characterized by GC-MS. HL compounds were categorized based on their characteristic functional groups by considering the reported methodology for other works in the literature [43]. The effect of reaction time and N_2_ purging conditions on the distribution of individual HL species is shown in Figure 3. Phenolics are the main compounds of HL (>65%), at all the operative conditions. Also, changing the reaction time and N_2_ feeding condition had no sequential effect on the overall distribution of HL species. However, it can be mentioned that by the change from batch to semi-batch condition, ethers percentage was increased; while by increasing the reaction time, ketones and aldehydes were increased.

**Figure 4:**
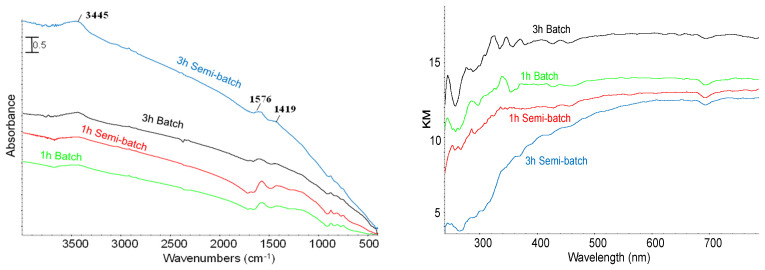
(**left**) FT-IR, and (**right**) UV-vis-NIR spectra of BCs.

BC samples were characterized by SEM-EDX (Table 2), FT-IR, and UV-vis-NIR spectroscopy (Figure 4). SEM-EDX results showed the presence of K and Ca which is typical for olive stone in agreement with literature data (CaO and K_2_O content of 14–33 wt.% and 2–32 wt.%, respectively) [41] and their presence could also be due to the eventual final treatments carried out in the industrial plant. In BCs, carbon and oxygen contents seem to be independent of applied pyrolysis conditions. However, a slight increase in carbon content in the absence of N_2_ flow (batch condition) could be due to a further decomposition of the tarry vapors onto the solid carbonaceous matrix through secondary reactions, in agreement with the literature [15].

FT-IR and UV-vis spectra of BCs, obtained from the different investigated conditions, showed similar features. These spectra are typical of highly carbonaceous solids, with few residual functional groups. Carbonaceous matrix could strongly absorb the IR radiation and cover the characteristic features of still incompletely converted biomass. However, FT-IR spectra show characteristic absorptions in the OH region (3600–3400 and 1700–1500 cm^−1^), due to adsorbed water on KBr, and a weak band of C−C deformation at 1420 cm^−1^ [1,7] and a complex feature in-between 1000–800 cm^−1^. The UV-vis-NIR spectra demonstrate full absorption of light in both the visible and UV range. Nevertheless, the BCs obtained under batch conditions display a higher level of light adsorption compared to those obtained under semi-batch conditions.

**Figure 5:**
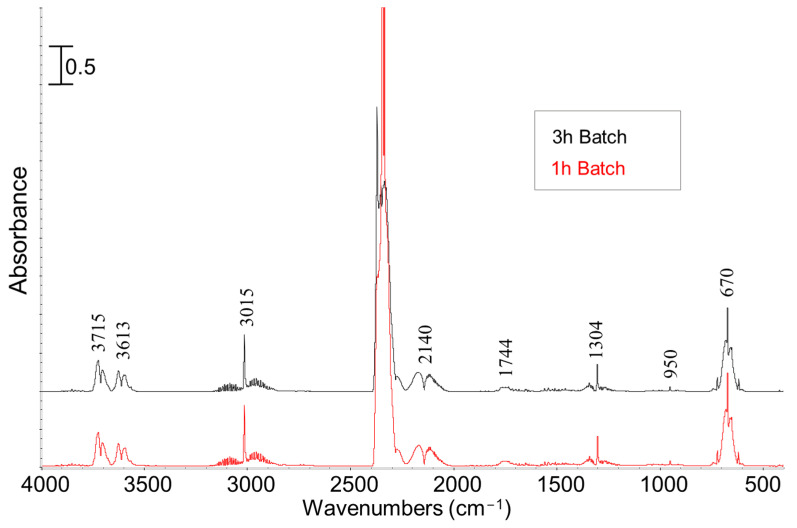
FT-IR spectra of PGs obtained from pyrolysis test carried out in batch condition.

PG have been characterized by means of GC-MS (Table 3) and FT-IR (Figure 5). It should be mentioned that only the PGs obtained from the pyrolysis test of batch condition have been collected and analyzed. The FT-IR spectra clearly showed the presence of carbon dioxide considering the bands centered at 670 cm^−1^ (bending), 2335 cm^−1^ (symmetric and asymmetric stretching), and corresponding bands at 3613 and 3715 cm^−1^ (overtones). The bands at 1304 cm^−1^ and 3015 cm^−1^ showed the presence of methane (bending and stretching rotovibrational modes, respectively). The weak bands at 2960 and 950 cm^−1^ are representative of C-H stretching of other hydrocarbons and CH_2_ out of the plane mode of ethylene (C_2_H_4_), respectively [44]. The band at 2143 cm^−1^ is due to the stretching mode of carbon monoxide. The band at 1744 cm^−1^ could be assigned to C=O stretching characteristic band [45].

In agreement with discussed IR data, GC-MS analysis revealed that the main PGs compounds are CO_2_, CO, CH_4_, and C_2_H_4_ as observed in literature where other types of biomasses such as *Arthrospira platensis* [1,3], wood, rice husk, and forestry residue [46] were used as raw feedstock. Moreover, no S-X vibration has been detected by considering the detectability limit and experimental errors. There were no significant differences observed in terms of PG compounds as the pyrolysis reaction time was increased from 1 to 3 h. It is worth mentioning that evaluating the impact of nitrogen feeding conditions, whether a continuous flow of N_2_ (semi-batch) or a batch N_2_ condition (0 mL min^−1^, batch), on the pyrolysis process, is crucial for process design and further possible applications of biomass.

### 2.3. Effect of Particle Size and Heating Rate

**Figure 6:**
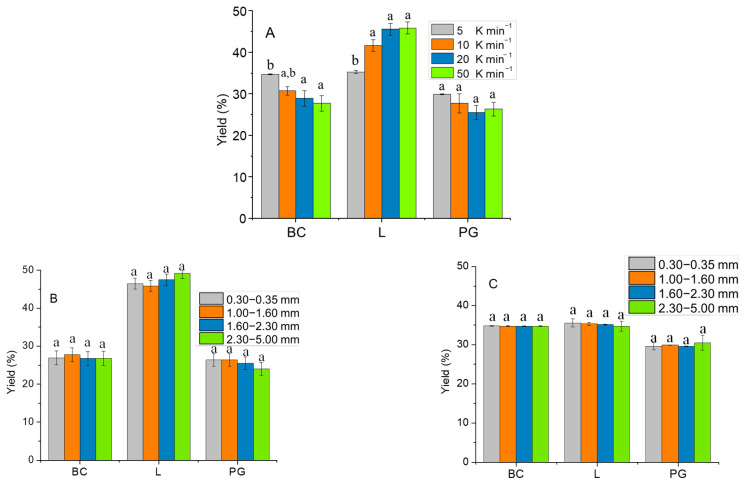
Effect of particle size and heating rate on pyrolysis products distribution, (**A**) variable heating rate and constant particle size (1.00–1.60 mm), variable particle sizes and constant heating rate of (**B**) 50 K min^−1^ and (**C**) 5 K min^−1^. Different letters (a, b) in the same group indicate statistically significant differences among mean values.

To ensure a more reliable comparison with the existing literature, a constant reaction time of 1 h under semi-batch conditions with constant nitrogen flow was upheld as a control in all experiments in this section, with only other parameters being altered [12,13,14,15,16]. This is significant as most of the related works in the literature utilize continuous carrier gas flow. The effect of changing the heating rate, by keeping a constant particle size (in the range of 1.00–1.60 mm), on the pyrolysis product distribution, is shown in Figure 6A. The BC and PG yield decreased while the L yield increased by increasing the heating rate, which is in agreement with literature reports [47,48]. In general, increasing the heating rate during biomass pyrolysis can result in higher yields of L and PG products, but can also lead to changes in the product composition and quality [14]. This behavior could be attributed to a quick biomass fragmentation and a higher tar decomposition increasing the heating rate. Biomass fragmentation refers to the breaking of a part of the solid biomass material into smaller pieces, which exposes more surface area to the heat source and allows for faster heating. Tar decomposition, on the other hand, refers to the breakdown of the tars and other volatile organic compounds that are released from the biomass during heating. The combined effect of quick biomass fragmentation and higher tar decomposition can lead to a significant increase in the heating rate, which can in turn affect the overall behavior of the system [31,49]. The fast biomass decomposition limits the available time for secondary reactions like tar cracking and re-polymerization that lead to the increase in liquids [50].

By maintaining a constant heating rate of 50 K min^−1^ (Figure 6B) while increasing the biomass particle size, the yields of L, PG, and BC remained constant based on statistical analysis (L followed a slight increasing pattern). The constancy of L yield at smaller particle sizes was surprising because smaller sizes typically result in higher heat transfer and greater biomass decomposition, leading to a higher liquid yield [51]. However, it was reported by Akhtar et al. [29] that factors such as biomass type, bulk density, and oxygen content can influence the trend between liquid yield and biomass particle size and therefore the results shown here are justified.

When increasing the biomass particle size with a constant heating rate of 5 K min^−1^ (Figure 6C), the distribution of BC, L, and PG was not affected at all. This can be attributed to the fact that at a slow heating rate, the temperature gradient between the particle surface and center is negligible, resulting in similar heat transfer rates for particles of different sizes.

**Figure 7:**
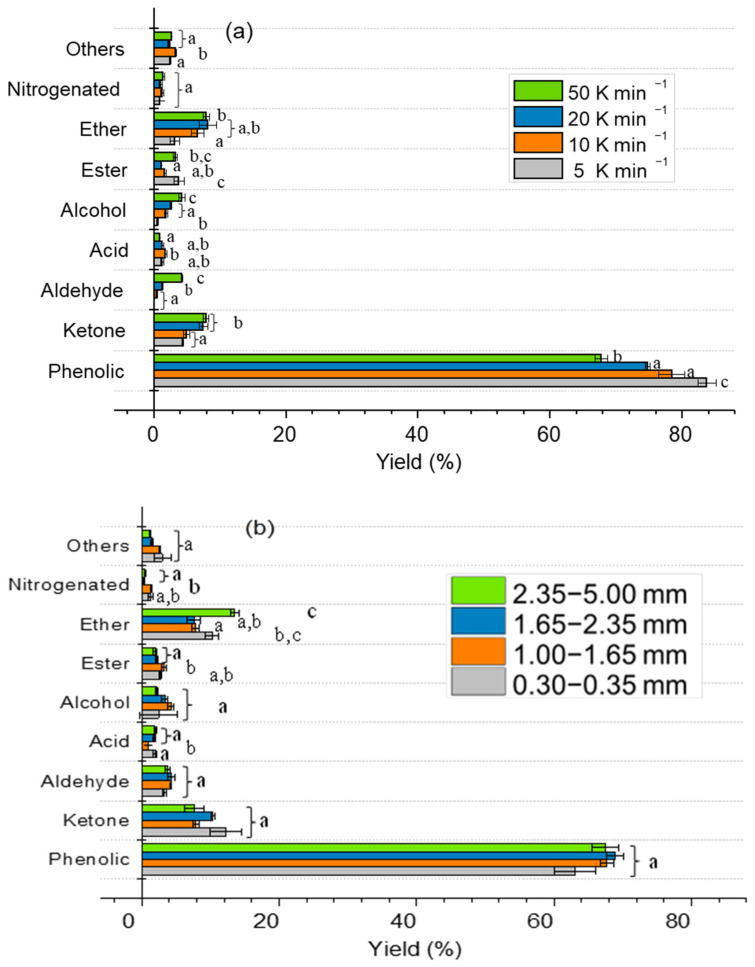

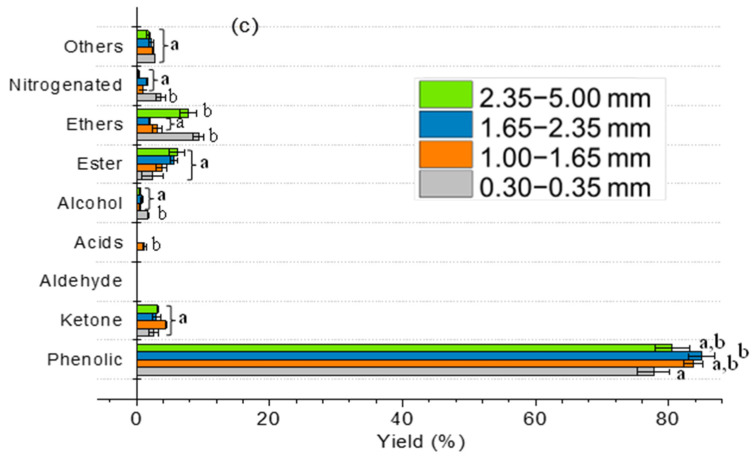
Effect of different pyrolysis conditions on the product distribution by GC-MS analysis, (**a**) variable heating rate and constant particle size of 1.00–1.60 mm, (**b**) variable particle sizes and constant heating rate of 50 K min^−1^, (**c**) variable particle sizes and a constant heating rate of 5 K min^−1^. Different letters (a–c) in the same group indicate statistically significant differences among mean values.

HL obtained from the pyrolysis condition with variable heating rate and constant particle size in the range of 1.00–1.60 mm have been analyzed with GC-MS and the results are shown in Figure 7. By increasing the heating rate, the phenolic content was decreased (maximum 20%). On the other hand, the content of aldehyde, alcohol, acetone, and ether groups was slightly increased. Additionally, the mass ratio between HL and LL was influenced by increasing the heating rate. The ratio of HL/LL at different heating rates of 5, 10, 20, and 50 K min^−1^ were 0.17, 0.39, 0.49, and 0.53, respectively. Therefore, higher heating rates produce a bio-oil richer in lipophilic compounds in agreement with [51]. The variation of biomass particle size with constant heating rate did not significantly influence the distribution and properties of obtained HL and LL.

**Figure 8:**
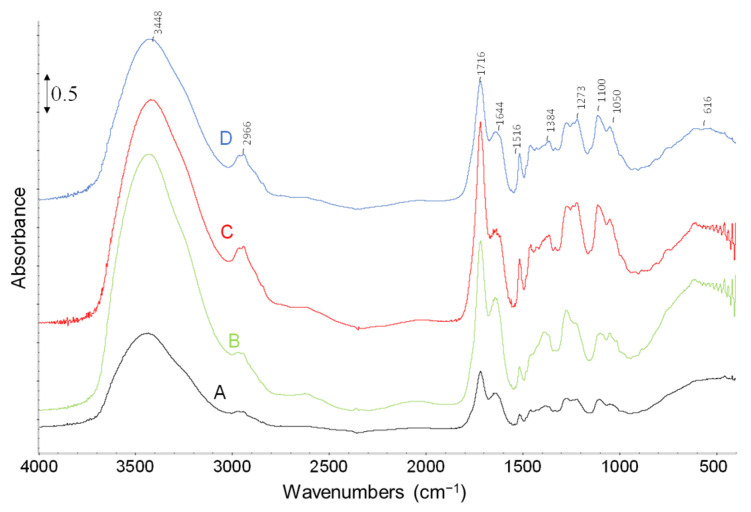
FT-IR spectra of LL samples obtained with biomass particle size in the range of 1.00–1.65 mm and heating rate of (A) 5 K min^−1^, (B) 10 K min^−1^, (C) 20 K min^−1^, (D) 50 K min^−1^.

Also, the higher heating rate resulted in darker LL and this trend was more obvious when the rate changed from 5 to 10 K min^−1^ while from 10 to 50 K min^−1^, the change in LL color was not clearly distinguishable. In Figure 8, the FT-IR analysis of LL obtained at various heating rates is reported, showing similar characteristic bands. The OH region exhibits characteristic absorptions at 3600–3400 cm^−1^ and 1680–1500 cm^−1^, assigned to moisture on KBr, while the CH stretching region shows absorptions at 3054–2959 cm^−1^. The C=O stretching of carbonyl, carboxyl, and ester groups exhibit a peak at 1716 cm^−1^ and a shoulder at 1800 cm^−1^. The bands at 1509 cm^−1^, 1466 cm^−1^, and 1426 cm^−1^ can be attributed to the C-C stretching of aromatics, whereas the 1384 cm^−1^ band is mainly associated with CH_2_ deformation. Furthermore, the 1331 cm^−1^ peak may be attributed to the -OH in-plane deformation of the phenolic group. The peaks in the 1000–1300 cm^−1^ range relate to various C-O and C-C coupling interaction stretching, while the peaks below 1000 cm^−1^ may be due to -OH and -CH out-of-plane deformation modes [1,7].

The results of SEM-EDX analysis for BCs produced by altering particle size and heating rate are presented in Table 4. The analysis revealed that there was no significant change in the elemental distribution of the BCs because of varying the particle size or heating rate. However, a lower carbon content was observed in the case of the smallest particle sizes (0.30–0.35 mm).

**Figure 9:**
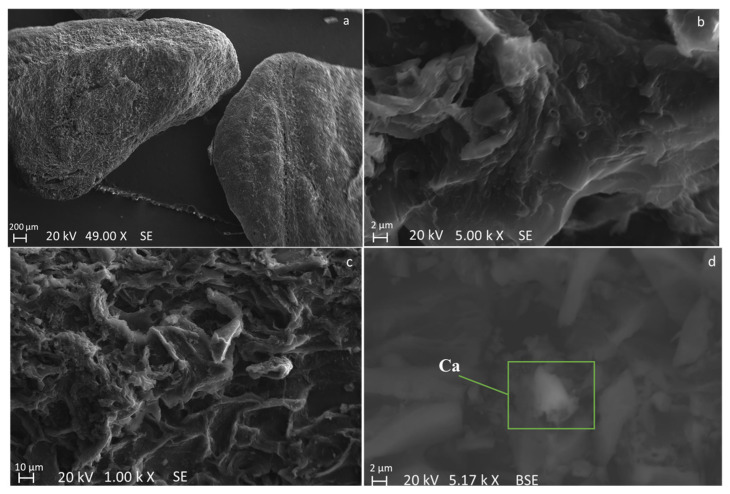
SEM Images of obtained BC obtained with particle size in the range of 1–1.65 mm and heating rate of 50 K min^−1^. Images are acquired by detecting different signals: secondary electron signal (**a**–**c**), and backscattered electron signal (**d**).

As an example, SEM micrographs (with different magnifications) of obtained BC with a particle size in the range of 1.00–1.65 mm and a heating rate of 50 K min^−1^ are reported in Figure 9. The SEM images show that the olive stone particles almost maintained their porous surface. However, it seems that the vascular fiber system (Figure 1a) is damaged and compacted to the external surface during pyrolysis (Figure 9a). Moreover, in Figure 9d, some agglomerated and non-homogenously distributed Ca particles are observable on the BC surface, as indicated by the brighter particles and confirmed by SEM-EDX point analysis. However, these inorganic agglomerated particles were not observed in the SEM images of the fresh biomass, even though some distributed brighter particles were envisaged at high magnification. The Ca-rich particles have also been observed in the BCs in other conditions. Therefore, it could be concluded that both agglomeration and accretion of Ca-rich particles occurred during the pyrolysis process [52].

**Figure 10:**
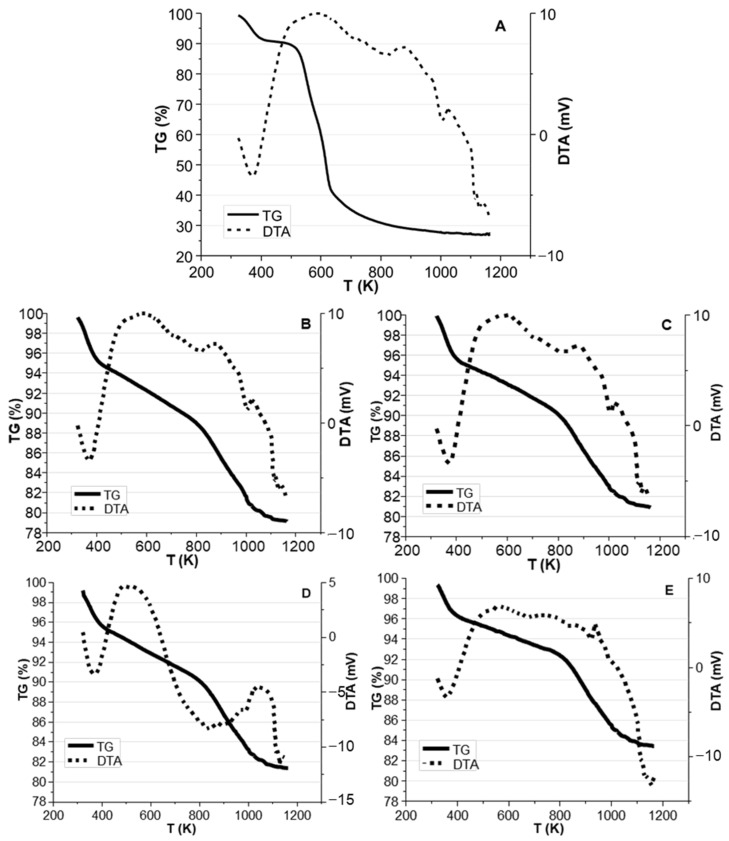
TG and DTA curves of (**A**) raw biomass, BC obtained with: (**B**) heating rate 50 K min^−1^ and particle size 1.00–1.65 mm, (**C**) heating rate 5 K min^−1^ and particle size 1.00–1.65 mm, (**D**) heating rate 50 K min^−1^ and particle size 2.30–5.00 mm, (**E**) heating rate 5 K min^−1^ and particle size 2.30–5.00 mm.

The thermogravimetric analyses of BC samples are shown in Figure 10. The first weight loss in all the samples occurred in the temperature range of 323–423 K due to free water evaporation. This could be confirmed by the endothermic peak at 373 K detectable in the DTA curves. For raw biomass (Figure 10A), a rapid weight loss is evident in the range of 473–823 K due to the decomposition of hemicellulose (493–588 K), cellulose (573–673 K), and lignin (423–1173 K) [53]. This could also be confirmed by the DTA exothermic peak centered at 623 K. Nassar et al. reported that in the case of using a heating rate of 10 K min^−1^, lignin decomposes very slowly, losing only 40% of its initial mass below 973 K [54]. Therefore, it could be concluded that the following decrease in biomass weight (~5%), in the range of 823–1173 K, is mostly due to the further decomposition of lignin. This could be confirmed by DTA peaks centered at 873, 1023, and 1123 K.

In the TGA and DTA analyses of BC samples (Figure 10B–E), and in the temperature range of 423 to 1073 K, two weight loss sections with different slopes are observable. The first one (range of 423–773 K) could be due to the desorption of surface-adsorbed compounds (~4%). In fact, considering that BCs are the solid product of pyrolysis, the weight loss could not be attributed to the degradation and decomposition of hemicellulose, cellulose, or lignin. The second part (range of 773–1173 K) is due to the decomposition of remaining lignin in the BCs. The weight loss behavior in the mentioned ranges is also confirmed by a broad exothermic peak in the DTA curves.

**Figure 11:**
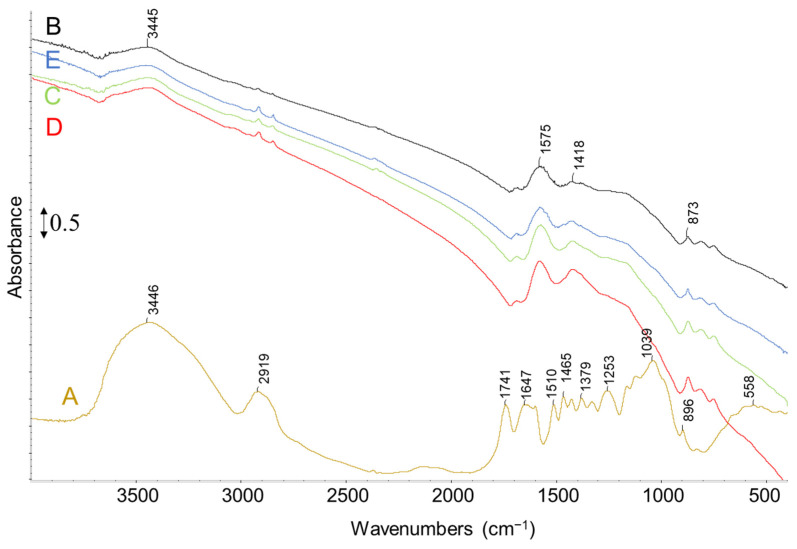
FT-IR spectra of (A) fresh olive stone, and BC obtained with biomass particle size 1–1.65 mm and heating rate of (B) 5 K min^−1^, (C) 10 K min^−1^, (D) 20 K min^−1^, and (E) 50 K min^−1^.

In this section, FT-IR spectra of fresh olive stone (Figure 11A) are reported to make a comparison with BCs. Spectra show several bands that are representative of various functional groups contained in the starting biomass. Also, it is comparable to the spectra of LL (Figure 8) which shows the presence of phenolic, alcoholic, carbonyl, and carboxyl groups together with water. In particular, the broad band centered at 3448 cm^−1^ is due to O-H stretching of water, phenolic and alcoholic groups. The band at 2918 cm^−1^ represents -CH stretching of aliphatics and aromatics while the band at 1736 cm^−1^ and its shoulder at 1800 cm^−1^ correspond to C=O bond stretching of carbonyl, carboxyl, and ester groups. The bands at 1600, 1509, 1466, and 1426 cm^−1^ can be related to C-C stretching of aromatics while the band at 1380 cm^−1^ is mainly related to CH_2_ deformation (scissoring). The -OH deformation modes of cellulosic units are also present in this region whereas the band at 1331 cm^−1^ could be due to -OH in-plane deformation of the phenolic group. The peaks in the range of 1000–1300 cm^−1^ are related to different C-O and C-C coupling interaction stretching while the peaks below 1000 cm^−1^ are likely due to -OH and -CH out-of-plane deformation modes [1,7].

FT-IR spectra of BC samples obtained with biomass particle size in the range of 1.00–1.65 mm at different heating rates (considering that the change in heating rate was the most effective parameter on the yield of BC) are shown in Figure 11. The obtained BCs present almost the same characteristic bands, like the one previously discussed (Figure 4). As expected, the characteristic bands observed in the spectra of fresh biomass have mostly disappeared which is due to the decomposition and carbonization of biomass by pyrolysis reaction.

Table 5 presents an overview of outcomes from some of the prior studies that focused on pyrolysis parameters and their impacts. However, given the diverse range of parameters, characterizations, operational setups, and conditions employed in these studies, making a direct comparison is unfeasible.

## 3. Materials and Methods

### 3.1. Materials

**Figure 12:**
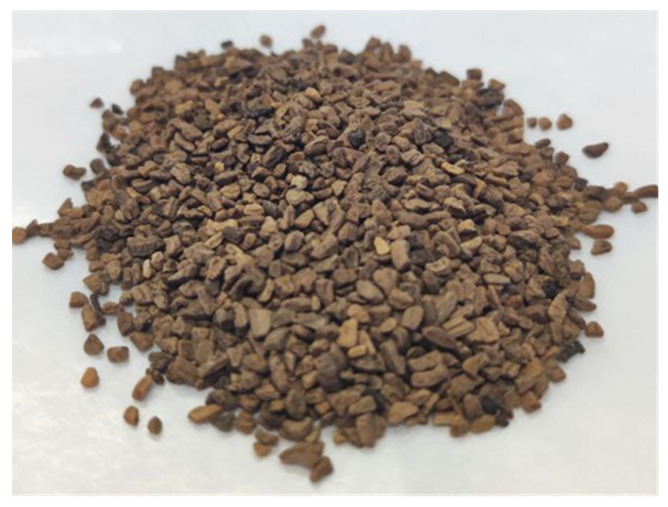
Industrial “end-of-life” olive stone waste biomass (endocarps).

In this work, end-of-life olive stones were used as the raw material, which was provided by a company located in Puglia, Italy. The olive stones displayed an overall particle size of 1–5 mm, and they were used without any chemical pretreatment but only by crashing and sieving operation (Figure 12). All the chemicals used in this work were provided by Carlo Erba reagents (Milan, Italy) with a purity above 99%.

### 3.2. Characterization of Biomass

Received biomass was characterized in terms of moisture and ash content, morphological and elemental composition, thermogravimetric analysis (TGA-DTA), and FT-IR (Nexus Thermo Fisher instrument, Madison, WA, USA) spectrophotometer. Moisture and ash content were measured in agreement with AOAC methods [7]. Morphological and elemental composition was investigated by a Zeiss Evo 40 equipped with a Pentafet Link Energy Dispersive X-ray Spectroscopy system managed by the INCA Energie 450 × 3 (Oxford Instruments, Analytical Ltd., Bucks, UK). Thermogravimetric analysis (TGA and DTA) was conducted using a TG-DSC Netzsch Geratebau STA 409 (Selb, Germany), equipped with a Netztch410 furnace temperature controller. In brief, about 60 mg of biomass was placed in a 0.1 cm^3^ alumina crucible and located inside the instrument in which the sample was flushed with nitrogen at a flow rate of 40 mL min^−1^, and then the temperature was raised from room temperature to 1173 K at a rate of 10 K min^−1^.

### 3.3. Pyrolysis Plant and Experimental Set-Up

**Figure 13:**
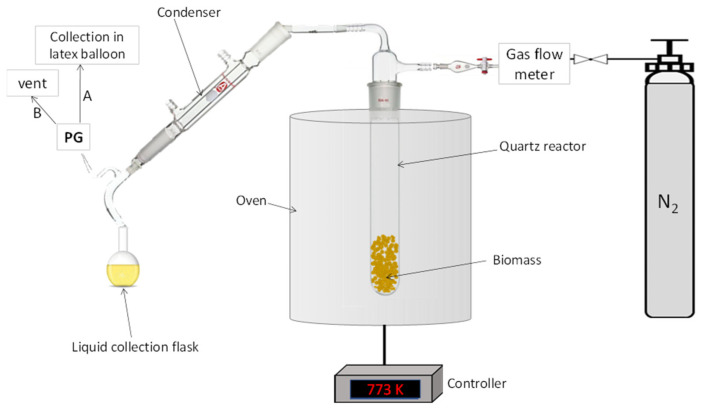
Scheme of the experimental setup used for the pyrolysis tests.

The thermal pyrolysis of olive stone endocarp particles was performed using a tubular quartz reactor within a vertical oven (Carbolite, MTF 10/25/130, Pocklington, UK). The olive stone particles were pre-dried in an oven for 24 h at a temperature of 378 K, following Ref. [56]. Afterward, the quartz reactor was charged with 10.00 g of dried biomass. The pyrolysis peak temperature (highest treatment temperature) for all the tests was 773 K, considered the optimal temperature for pyrolysis based on various reports (generally pyrolysis can be conducted in a range of 623–923 K) [50,53,57,58,59,60]. The reaction system was connected to a nitrogen line (N_2_, Alphagaz 1, Air Liquide, Milan, Italy) providing an oxygen-free atmosphere. The reactor was connected to a Liebig condenser to separate the condensable vapors as a liquid from the gaseous products. A flask was used to collect the L products. Collected L was made up of two phases: a water-soluble portion at the top layer, named LL, and a viscous mostly oligomeric lignin-derived fraction settled at the bottom, labeled as HL. Non-condensable gases, named PG, were collected in a latex balloon (in batch condition) or vented (in semi-batch condition). The solid residue, BC, was collected inside the reactor. A schematic of the reaction system is shown in Figure 13.

For a systematic comparison, two different experimental conditions have been investigated, as described in the following sections. A repeatability test for pyrolysis was conducted to evaluate the percentage error in the product yield. The GC-MS analysis was performed in duplicate, and the values are expressed as average, while the error is expressed as the standard deviation.

#### 3.3.1. Investigation of the Effect of Reaction Time and Nitrogen Purging Conditions

Thermal pyrolysis on olive stone particles (sized 1–5 mm) using a heating rate of 50 K min^−1^ for 1 or 3 h was performed. Tests have been performed in an O_2_-free environment in the presence of a continuous N_2_ flow (100 mL min^−1^, semi-batch) or in batch N_2_ (static atmosphere). Therefore, the effect of reaction time and nitrogen purging conditions on the distribution and composition of the products have been investigated.

#### 3.3.2. Investigation of the Effect of Biomass Particle Size and Heating Rate

Thermal pyrolysis with a reaction time of 1 h in the presence of 100 mL min^−1^ of N_2_ flow with different biomass particle sizes in the ranges of 0.30–0.35, 1.0–1.6, 1.6–2.4, and 2.4–5.0 mm and different heating rates, i.e., 5, 10, 20, and 50 K min^−1^ were investigated.

### 3.4. Characterizations of Pyrolysis Products

All the reaction products (BC, L, PG) were analyzed by Fourier Transform Infrared Spectroscopy (FTIR) using a Nicolet 380 FT-IR Spectrometer (Thermo Scientific, Madison, WA, USA). All the spectra were elaborated using Omnic Lite Software version 5.12 (Thermo Electron Corporation, Madison, WA, USA). To analyze the solid product (BC), samples and KBr were mixed (1 wt.% BC) and pressed, while L samples were dispersed on a KBr-pressed disk. PG samples were injected into an IR glass cell with KBr windows and analyzed by the same spectrometer. HL and PG samples were also analyzed by a gas chromatograph-mass spectrometer (GC-MS) Focus-ISQ supplied with a single quadrupole detector (Thermo Scientific, Milan, Italy) using a TG-SQC column (30 m × 0.25 mm × 0.25 µm) using He (>99.99) as carrier gas. To analyze L, the HL phase was diluted with CHCl_3_ with a ratio of 1:10 (*v*/*v*) and then 1 µL of the diluted L was injected into the GC-MS by using a 10 µL syringe. To analyze PG, 100 µL of the sample was injected into the GC-MS using the column mentioned above. The areas of the resulting peaks related to the individual species were normalized with the area of the total peaks to calculate the relative amount of the various species in the sample. Moreover, the yield of the products and distribution of HL were analyzed by Statistica software^®^ version 12 by ANOVA method.

Additionally, BC samples were analyzed by UV-vis-NIR spectroscopy by means of a V570 instrument (JASCO Corp., Tokyo, Japan) and Thermogravimetry (TGA and DTA) in the same conditions as those reported in Section 3.2. For the evaluation of BC morphology and composition, Scanning Electron Microscopy (SEM) and Energy Dispersive X-ray spectroscopy (EDX) have been used.

## 4. Conclusions

In this study, industrial end-of-life olive stone waste was chosen as the feedstock for pyrolysis to examine the impact of key operational parameters on the pyrolysis products and provide a comprehensive comparison. Results showed that changing the reaction time from 1 to 3 h and using N_2_ feeding conditions, either batch or semi-batch, did not significantly affect the product distribution and composition. The obtained L, accounting for approximately 45 wt.%, comprised two phases with a heavy-to-light liquid ratio of 0.53. Phenolic compounds made up over 65% of the HL products. The PG, accounting for approximately 28 wt.%, was primarily composed of H_2_, CO_2_, CO, CH_4_, and C_2_H_4_. BC, accounting for approximately 28 wt.%, was also produced. Increasing the heating rate while keeping the olive stone particle size constant, affected both distribution and composition of the obtained products. A decrease in BC yield of about 20% and an increase in L yield of about 22% were observed with higher heating rates. Additionally, the HL to LL ratio increased from 0.17 to 0.53, while the phenolic group in the heavy fraction decreased. However, no significant changes in BC properties were detected. On the other side, changing the olive stone particle size while keeping the heating rate constant had a negligible impact.

In conclusion, olive stone has the potential as a precursor for developing carbon-based materials. The gas produced from olive stone pyrolysis can be used for further processing to produce syngas and chemicals like methanol. However, the L produced from the pyrolysis is dominated by oxygenated functional groups, leading to low energy density and instability. Therefore, further upgrading is necessary to convert the L into useful chemicals or combustion fuel. This study provides a deeper understanding of the relationship between pyrolysis parameters and the resulting products.

## Figures and Tables

**Table 1:** SEM-EDX global composition of fresh biomass and ashes. Minimum detection limit ~0.1 wt.%.

Sample	C (wt.%)	O (wt.%)	Na (wt.%)	K (wt.%)	Ca (wt.%)	Mg (wt.%)	Si (wt.%)	P (wt.%)	S (wt.%)
Fresh Biomass	53	47	n.d.	n.d.	n.d.	n.d.	n.d.	n.d.	n.d.
Ash	5.5	51.5	1.4	24.0	12.8	2.0	1.0	0.8	1.0

n.d. not detectable.

**Table 2:** SEM-EDX global composition of BC in different pyrolysis conditions (change in reaction time and N_2_ gas).

Biomass Size	Heating Rate	Reaction Time (h)	N_2_ Condition	C (%)	O (%)	K(%)	Ca (%)
1–5 mm	50 K min^−1^	3	Batch	82.5	15.5	1.5	0.5
		3	Semi-batch	81.5	15.5	1.9	1.1
		1	Batch	84.0	14.5	1.5	0.0
		1	Semi-batch	81.0	16.5	1.5	1.0

**Table 3:** The product distribution of PG determined by GC−MS in batch conditions.

Compound	Distribution (%)	
	3 h without N_2_	1 h without N_2_
N_2_, H_2_, CO_2_, CO, CH_4_, C_2_H_4_	77.49 ± 0.81	74.06 ± 0.74
Acetaldehyde & 2-Butene	6.79 ± 0.25	8.76 ± 0.76
Formic acid, methyl ester	2.04 ± 0.70	2.27 ± 0.05
1-Butane, 2-methyl-	0.03 ± 0.02	0.06 ± 0.02
Furan & Acetone	4.63 ± 0.15	4.57 ± 0.06
1-Butan, 3-methyl-	0.56 ± 0.05	0.31 ± 0.06
Acetic acid, methyl ester	1.58 ± 0.07	1.55 ± 0.09
Cyclopropane, ethynyl-	0.67 ± 0.00	0.47 ± 0.04
Propanal, 2-methyl-	0.34 ± 0.03	0.39 ± 0.01
2,3-Dihydrofuran	0.58 ± 0.04	1.40 ± 0.04
2,3-Butanedione	0.75 ± 0.06	1.41 ± 0.08
2-Butanone	0.42 ± 0.03	0.42 ± 0.01
Furan, 2- or 3-methyl-	3.42 ± 0.09	3.54 ± 0.02
Methyl propionate	0.12 ± 0.02	0.14 ± 0.01
1,4-Cyclohexadiene	0.11 ± 0.01	0.09 ± 0.02
2,4-Hexadiene	0.11 ± 0.01	0.13 ± 0.02
Benzene	0.16 ± 0.04	0.17 ± 0.02
Furan,2,5-dimethyl	0.21 ± 0.01	0.24 ± 0.03

**Table 4:** SEM-EDX global composition of BCs obtained by changing the particle size and heating rate of pyrolysis tests.

Biomass Size (mm)	Heating Rate (K/min)	C (%)	O (%)	K (%)	Ca (%)
1.00–1.60	5	81.4	16.4	1.4	0.8
	10	82.0	15.6	1.4	1.0
	20	82.5	14.0	1.6	1.9
	50	79.5	17.5	1.5	1.5
1.60–2.30	50	83.0	14.4	1.8	0.8
2.30–5.00		83.0	14.5	1.7	0.8
0.30–0.35		77.5	17.5	2.2	2.8

**Table 5:** Presents an overview of outcomes from some of the prior studies that focused on pyrolysis parameters and their impacts.

Biomass	Pyrolysis Condition	Products Yield (wt. %)	Main PG Composition (Order Based on Abundance)	Main L Composition	BC Analysis	Ref.
Wood	Fast, at 1123 K, with 300–500 K s^−1^, static N_2_	PG ≅ 78 L ≅ 12 BC ≅ 10	CO, H_2_, CO_2_, CH_4_	n.a.	n.a.	[55]
	Slow, at 1123 K, with 10 K m^−1^, for 30 min, static N_2_	PG ≅ 23 L ≅ 61 BC ≅ 16	CO, CO_2_, H_2_, CH_4_			
Wheat straw	Slow, at 823 K, with 5 K m^−1^, for 60 min, atm of N_2_ with residence time of 100 s, pressure of 0.2 MPa	PG ≅ 33 L ≅ 39 BC ≅ 28	CO_2_, CO, H_2_, CH_4_	y_org_:y_water_ = 0.27 Detailed analysis n.a.	H:C ≅ 0.37 O:C ≅ 0.08 S_BET_ = 229 m^2^g^−1^ V_ultra_ = 0.08 cm^3^g^−1^	[13]
	Slow, at 748 K, with 5 K m^−1^, for 60 min, atm of CO_2_/N_2_ = 0.3 with residence time of 150 s, pressure of 0.55 MPa	PG ≅ 36 L ≅ 35 BC ≅ 29	CO_2_, CO, CH_4_, H_2_	y_org_:y_water_ = 0.43 Detailed analysis n.a.	H:C ≅ 0.46 O:C ≅ 0.11 S_BET_ = 203 m^2^g^−1^ V_ultra_ = 0.05 cm^3^g^−1^	
Cotton seed cake	At 823 K, with 300 K min^−1^, for 10 min, N_2_ of 50, 100, 200, 400 cm^3^min^−1^	For N_2_ of 100 cm^3^min^−1^ PG ≅ 30 L ≅ 44 BC ≅ 26	n.a.	For N_2_ of 100 C ≅ 70% O ≅ 16% N ≅ 6% H ≅ 9%	n.a.	[47]
	At 823 K, with 5, 100, 300, 700 K min^−1^, for 10 min, N_2_ of 100 cm^3^min^−1^	For rate of 5 K min^−1^ PG ≅ 21 L ≅ 47 BC ≅ 32		For rate of 300 K min^−1^C ≅ 70% O ≅ 16% N ≅ 6% H ≅ 9%		
Vine shoots	At 873 K, with 5 K min^−1^, for 60 min, static N_2_, pressure of 0.1 MPa	PG ≅ 23 L ≅ 44 BC ≅ 33	CO_2_, CO, CH_4_, H_2_	n.a.	H:C ≅ 0.40 O:C ≅ 0.07 S_BET_ = 109 m^2^g^−1^	[25]
	At 873 K, with 5, K min^−1^, for 60 min, static CO_2_, pressure of 0.1 MPa	PG ≅ 27 L ≅ 43 BC ≅ 30	CO_2_, CO, CH_4_, H_2_		H:C ≅ 0.38 O:C ≅ 0.05 S_BET_ = 57 m^2^g^−1^	
Olive husk	At 950 K, with 10 K s^−1^, biomass size of <0.5, 1.0, 1.5, 2.0 mm	0.5 mm BC ≅ 18 1.0 mm BC ≅ 24 1.5 mm BC ≅ 29 2.0 mm BC ≅ 36	n.a.	n.a.	n.a.	[21]
Olive stone	Slow, at 773 K, with 50 K min^−1^, biomass size of 1–5 mm, for 60 or 180 min, static N_2_ or a flow of 100 mL min^−1^	For 60 min and static N_2_ PG ≅ 27 L ≅ 47 BC ≅ 26	CO_2_, CO, H_2_, CH_4_, C_2_H_4_	Phenolics ≅ 68% Ketones ≅ 5% Ethers ≅ 10% Alcohols ≅ 8% Ester ≅ 6%	C ≅ 84.0% O ≅ 15.5% K ≅ 1.5% Ca ≅ 0.5% H = n.a.	This work
	Slow, at 773 K, with 5, 10, 20, 50 K min^−1^, biomass size of 0.30–0.35, 1.0–1.6, 1.6–2.4, and 2.4–5.0 mm, for 60 min, N_2_ flow of 100 mL min^−1^	For 5 K min^−1^ and 1.0–1.6 mm PG ≅ 30 L ≅ 35 BC ≅ 35	n.a.	Phenolics ≅ 84% Ketones ≅ 6% Ethers ≅ 4% Acids ≅ 2% Ester ≅ 5%	C ≅ 81.4% O ≅ 16.4% K ≅ 1.4% Ca ≅ 0.8% H = n.a.	

n.a. not available.

## Data Availability

Data will be available on request.
